# Temporal phase evolution OCT for measurement of tissue deformation in the human retina *in-vivo*

**DOI:** 10.1364/BOE.440893

**Published:** 2021-10-25

**Authors:** Sylvia Desissaire, Florian Schwarzhans, Stefan Steiner, Clemens Vass, Georg Fischer, Michael Pircher, Christoph K. Hitzenberger

**Affiliations:** 1 Center for Medical Physics and Biomedical Engineering, Medical University of Vienna, Vienna, 1090, Austria; 2 Center for Medical Statistics, Informatics and Intelligent Systems, Medical University of Vienna, Vienna, 1090, Austria; 3 Department of Ophthalmology and Optometry, Medical University of Vienna, Vienna, 1090, Austria

## Abstract

We demonstrate the use of temporal phase evolution (TPE-) OCT methods to evaluate retinal tissue deformation *in-vivo* over time periods of several seconds. A custom built spectral domain (SD)-OCT system with an integrated retinal tracker, ensuring stable imaging with sub-speckle precision, was used for imaging. TPE-OCT measures and images phase differences between an initial reference B-scan and each of the subsequent B-scans of the evaluated temporal sequence. In order to demonstrate the precision and repeatability of the measurements, retinal nerve fiber (RNF) tissue deformations induced by retinal vessels pulsating with the heartbeat were analyzed in several healthy subjects. We show TPE maps (M-scans of phase evolution as a function of position along B-scan trace vs. time) of wrapped phase data and corresponding deformation maps in selected regions of the RNF layer (RNFL) over the course of several cardiac cycles. A reproducible phase pattern is seen at each heartbeat cycle for all imaged volunteers. RNF tissue deformations near arteries and veins up to ∼ 1.6 µm were obtained with an average precision for a single pixel of about 30 nm. Differences of motion induced by arteries and veins are also investigated.

## 1. Introduction

Investigation of the relations between changes in retinal structural and biomechanical properties and various diseases has been of increasing interest over the last decades. A large number of studies reported on the prospects of identifying retinal alterations in patients with degenerative or other cerebrovascular and cardiovascular diseases to define new biomarkers in order to improve clinical screening and monitoring [1–5]. Compared to other organs of the body, the eye has indeed the substantial advantage of being easily and non-invasively accessible *in-vivo* using optical coherence tomography (OCT). This imaging technique is already a gold standard in ophthalmology for the diagnostics of ocular diseases. While the different retinal layers are visualized in OCT intensity images, the phase data provide additional valuable information, especially for the analysis of the retinal vasculature. In several clinical applications, OCT angiography (OCTA), an extension of OCT, is increasingly used to examine the retinal vascular network [6]. In this label free method, the vascular information can be accessed either from the intensity and/or phase data by differentiating between static tissues and moving red blood cells. Doppler OCT, another extension of OCT, additionally allows to quantify blood flow from the phase difference between successive A-scans [7].

Phase-sensitive OCT (PhS-OCT) is not only a valuable technique to study fast motion in the vasculature itself but may also be applied for an analysis of slower rate length changes at the nanoscale in the eye [8,9] or for an investigation of tissue deformation in other tissues and organs [10–12]. PhS methods are commonly used in optical coherence elastography (OCE) as well, another extension of OCT that additionally provides access to tissue biomechanical properties, especially the tissue elasticity [13,14]. In OCE, tissue motion is usually accessed by determining differences in the amplitude and/or the phase between consecutive A-scans or B-scans [15–17] and inter-frame deformation can directly be obtained using the finite difference method (FDM) [18]. Phase gradient methods, that are based either on the least square method (LSM) [18] or on the vector method (VM) [19], are however more commonly applied for the estimation of the inter-frame strain in OCE, as the sensitivity of such methods is higher compared to the FDM [18]. Depending on the motion rate, unwrapping, which may be subject to error, may be required for most phase sensitive methods prior to strain estimation. This step is avoided in the VM as complex-valued signals are considered. The cumulative strain relative to the initial frame can finally be obtained by summation of the individual inter-frame data. The estimation of cumulative strain by such incremental method has been further optimized for slow rate tissue deformation imaging, as shown in recent studies [20,21]. In these works, rather than performing strain calculation for each pair of consecutive scans, the inter-frame time interval is adjusted according to the speed of the deformation. Another method used for the evaluation of tissue motion is based on the calculation of relative phase difference over time between two retinal layers and to compare this difference to the value of a fixed initial volume, as demonstrated for functional imaging in the retina using a full field (FF-) swept-source (SS-) OCT system [9,22]. The total issue displacement is then retrieved after temporal integration of the phase difference data. In non-moving (e.g. excised) tissue, a fixed reference is sometimes chosen for the phase difference calculation, as is done for example between A-scans in photo-thermal OCT [23].

Various studies investigated the use of PhS-OCT and OCE methods for ophthalmic applications. In OCE, investigations on corneal imaging, either *in-vivo* in animal models or *ex-vivo* [17,24–27] have mostly been reported. In recent years, *in-vivo* human corneal OCE imaging was also demonstrated [28–30], enabling to calculate the elastic modulus in human subjects *in-vivo* which was not possible with other existing techniques probing the mechanical properties of the cornea [24]. As non-invasive OCE measurements are challenging, reliable quantitative evaluation for clinical translation is up to now still limited [31]. OCE measurements in retinal tissue pose even greater challenges, and only *ex-vivo* samples [32,33] as well as *in-vivo* animal models [34] have so far been imaged. While full elastography requires information on the force exerted on the tissue, which is difficult to access *in-vivo*, passive elastography is a promising alternative technique used to access biomechanical properties of tissues without the need of an active external source. Passive excitation can result from the cardiac beatings or muscle contractions, among other examples. *In-vivo* OCT investigations in animal models have already shown the potential of the method for the evaluation of both corneal stiffness [35] and corneal tissue response [36] under natural pulsatile motion. While the first study focuses on the analysis of mechanical shear wave propagation, the second study is based on the calculation of tissue deformation and strain using the LSM over time. In-vivo heartbeat induced corneal strain measurements were also recently demonstrated in human eye using ultrasound elastography [37]. Similarly, several human *in-vivo* studies using PhS-OCT also focused on the investigation of deformation in corneal or retinal tissue under pulsation. Spahr et al. have shown retinal nanoscale tissue motion in the surrounding of retinal vessels over several cardiac cycles imaged with a FF-SS-OCT [38] (using the phase difference method [9,22]). Measurements of the trabecular meshwork motion [39,40] and the lamina cribrosa motion [41] under pulsation, particularly of interest for glaucoma research, were also conducted using PhS- spectral domain (SD-) OCT systems. In these two studies, tissue motion was retrieved from the phase difference calculated between adjacent B-scans only, providing therefore information on tissue deformation that is large and fast enough to induce measurable phase changes on a short time scale in the range of milliseconds.

Successful investigation of tissue deformation in the eye, either with OCT or OCE, is highly dependent on phase stability. Different optimization approaches to achieve higher phase stability have been investigated by, for example, studying the instabilities of swept sources [42], increasing the imaging speed [43] or testing common path configurations [17,44]. In ophthalmic applications, the use of real time eye trackers, usually employing a scanning laser ophthalmoscope (SLO) integrated to an OCT system, has also greatly improved the stability of *in-vivo* measurements. Different tracking techniques have been implemented both in commercial and research OCT instruments, as seen in the literature [45–47]. Such technical improvements have fostered the use of OCT imaging for new applications.

In this work, we present the results of imaging and measuring deformation induced in the retinal nerve fiber layer (RNFL) by retinal vessels pulsating with the heartbeat in healthy human subjects. Measurements were done using a custom-built SD-OCT system with an integrated retinal tracker that stabilizes imaging with sub-speckle precision. To detect the subtle deformation induced changes, the phase information provided by OCT is used. Two methods were used for the evaluation of phase differences over time, both providing access to the temporal phase evolution (TPE) of data over several seconds. The phase data are converted into displacement data and displayed as maps that show tissue displacement vs. time at a given depth and lateral position within tissue. Thereby, tissue deformation on the scale of a few nm and over extended time periods of several seconds is demonstrated. We also analyze differences in tissue deformation induced by arteries and veins and report on the repeatability and precision of the method.

## 2. Methods

### 2.1 Temporal phase evolution OCT

**Fig. 1.**
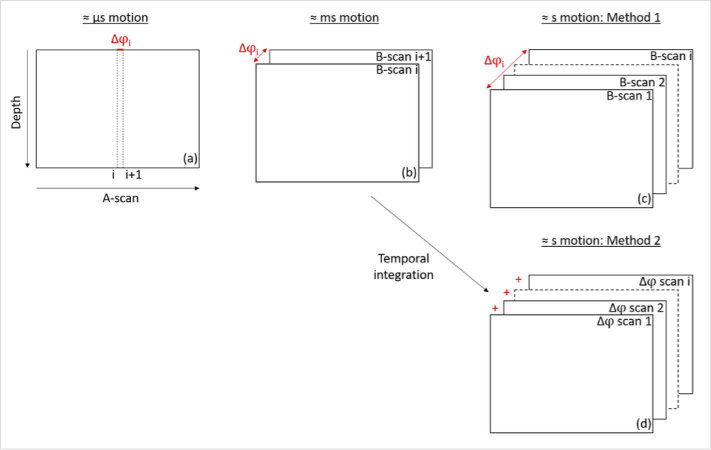
Comparison between the TPE OCT methods (M1 and M2) used to access motion in the range of seconds and the usual PhS methods used for example in standard Doppler OCT and OCTA to evaluate motion in the range of microseconds and milliseconds, respectively.

In this study, we aim to measure sub-micron tissue motion over an extended timeframe, i.e. over several seconds. OCT based motion measurements typically compare phase signals recorded at the same position with a time lag Δτ between them. Depending on the motion speed to be measured, and depending on whether absolute or relative motions are to be analyzed, different evaluation strategies are used. The phase calculation can either be performed between A-scans (typically used for Doppler OCT [7], Fig. 1(a)), between B-scans (typically used for OCTA and also for OCE [24,48], Fig. 1(b)), or between volumes, and may consider a fixed reference, a moving reference, or referencing to all other frames simultaneously (as proposed by the extended Knox Thompson algorithm [49,50]). The standard Doppler method is not sensitive to bulk motion because it uses time lags in the order of ∼ 1–100 µs and therefore is only sensitive to rather fast motions that occurs in large retinal vessels. However, the range of measureable velocities can be increased using different scanning protocols as demonstrated in [51]. In OCTA, Δτ ∼ 1–100 ms is typically used for contrasting slow motion within capillaries This method is however more sensitive to both axial and lateral bulk motions, and quantitative information on slowly moving tissue components is difficult to achieve.

**Fig. 2.**
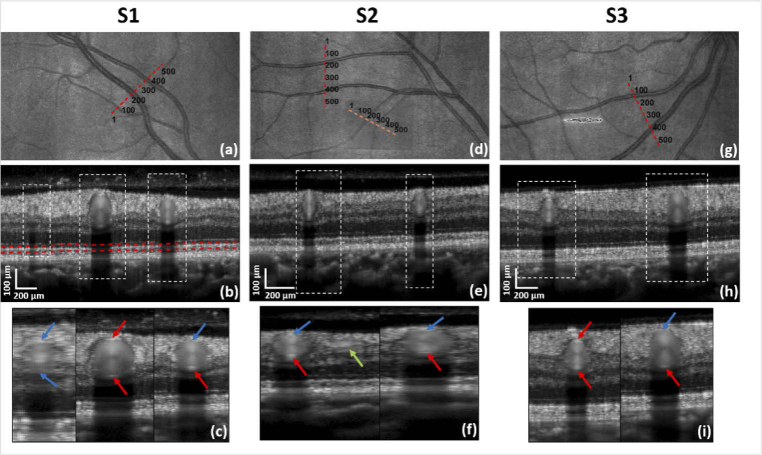
Overview of B-scans and their locations for the three study subjects. (b), (e) and (h) are the averaged intensity images of the traces marked by red dotted lines on the LSLO images (a), (d) and (g) for subjects S1, S2 and S3, respectively. The diameter of the vessels is calculated between the vessel walls as marked by the arrows in the zoomed images (c), (f) and (i). Some brighter walls, marked in red, can be more easily identified than others, marked in blue. The trace marked in orange in (d) is shown in Fig. 6.

In order to analyze slower motion happening over several seconds, such as tissue deformation, we study the evolution of the optical phase signal over time with respect to a fixed reference frame (for which we introduce the term TPE-OCT). We used two different methods that also provide information on absolute tissue positions relative to a starting point. In the first method (M1), the phase differences between a reference B-scan and each of the subsequent B-scans of the acquired sequence, providing a Δτ of ∼ 0.1–10 s (Fig. 1(c)), are calculated. As the SD-OCT system used for imaging has an integrated retinal tracker providing sub-speckle precision [46,52] (c.f. the preserved speckle structure in the averaged B-scans of Figs. 2(b), (e), (h)), the impact of lateral motions is greatly reduced in our measurements. In the second method (M2), the phase difference between consecutive B-scans is calculated followed by temporal integration relative to an initial reference frame. This second method has been used for example, in OCE and FF-OCT [36,38]. If phase unwrapping is performed for the data provided by M1, both methods are mathematically equivalent [53], however, each of them has slight advantages for some specific data presentation schemes, so we will demonstrate results obtained with both of them (in the majority of the presented results, we used M1).

### 2.2 Measurement protocol

#### 2.2.1 Experimental set-up

A custom built SD-OCT with an integrated retinal tracker is used for imaging [52]. It operates with a superluminescent diode (SLD) with a center wavelength of 860 nm and a bandwidth of 60 nm at an A-scan rate of 70 kHz and has a sensitivity of 98 dB. The resolution achieved is 4.2 µm axially (in tissue) and 20 µm laterally. The retinal tracker, essential feature for the purpose of this study, is composed of a line SLO (LSLO) with a laser light source at a center wavelength of 786 nm. A GPU-based algorithm is used for real time tracking as described in previous literature [46]. The power to the eye is 0.7 mW from the LSLO and 0.5 mW from the OCT beam, which satisfies the laser safety regulations.

#### 2.2.2 Acquisition procedure

We are interested in measuring RNF tissue movement induced by retinal vessels that pulsate with the heartbeat, and to analyze the differences between deformation caused by the veins and the arteries. For each subject, we thus chose to acquire cross sections of RNF tissue in the vicinity of retinal vessels, including at least one artery and one vein. The B-scan traces, which can be of arbitrary orientation, were manually selected before imaging on the template LSLO image as shown in Figs. 2(a), (d), and (g). The coordinates of the selected points were then converted to input amplitudes for the x and y OCT scanners after linear interpolation at evenly spaced positions along the trace, as described in [54]. In order to obtain images with sufficient RNFL thickness and rather large vessels, ocular fundus regions nasally inferior or superior to the macula were preferably chosen. The lengths of the selected traces were about 1.6 to 2.2 mm and were adjusted to include sufficient RNFL tissue around the vessels while keeping a rather high sampling density and minimizing the effect of head rotation that will be more pronounced for longer traces. For each measurement, 1000 B-scans of 512 A-scans were recorded at the same position corresponding to an acquisition sequence of about 10 s.

#### 2.2.3 Subjects’ selection

Three eyes of three healthy volunteers (aged 30, 34 and 32, respectively S1, S2 and S3) were included in this analysis. The study was approved by the university’s ethics committee and are in agreement with the tenets of the Declaration of Helsinki. The averaged intensity images of the traces as well as their location on the LSLO are shown in Fig. 2.

### 2.3 Data processing

#### 2.3.1 Standard processing and registration

**Fig. 3.**
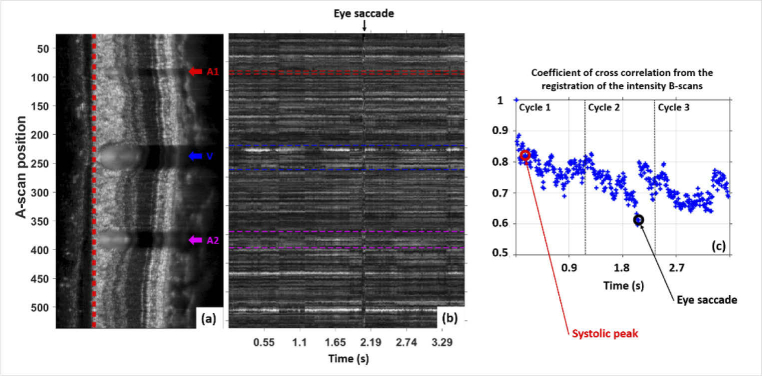
Illustration of image registration results. (a) Averaged intensity B-scan, covering three cardiac cycles, after registration and flattening. Two arteries (A1, A2) and one vein (V) are marked by arrows. (b) M-scan (vertical: distance along B-scan trace; horizontal: time) of the top surface of the RNFL. (c) Cross-correlation coefficient of intensity B-scans after registration (with respect to reference frame). The red circle marks the systolic peak at which images of Fig. 4 (a)-(c) are taken, the black circle marks the position where Fig. 4 (d) is taken.

Standard Fourier Domain (FD-) OCT processing of the raw data was used to obtain the individual 1000 intensity and phase B-scans for each recorded data set. In order to correct for axial and lateral motions, registration was performed on the intensity scans, taking the first intensity B-scan as reference and using a similar method as described in [55]. The phase B-scans were registered by using the same shifts as for the intensity scans. Figure 3(a) shows an averaged intensity B-scan image after registration and flattening (with reference to the anterior RNFL surface), covering three cardiac cycles. The positions of two arteries and one vein are marked by arrows. The preserved speckle pattern provides a first impression of the high quality of tracking and registration. Figure 3(b) shows an M-scan (distance along B-scan trace vs. time) of the top boundary of the RNFL over time. Straight horizontal lines indicate individual bright speckles over time. Apart from a saccadic eye movement, the maximum lateral shift after registration was ∼ 2 pixels. Figure 3(c) shows the cross-correlation coefficient of each intensity B-scan with respect to the reference frame after registration. Values of ∼ 0.7 or larger can be considered as good.

The diameter of each vessel was extracted from the averaged intensity scan, considering the number of pixels between the upper wall and lower wall in depth (see the arrows in Figs. 2(c), (f) and (i)). As the vessels may not be imaged perfectly perpendicularly and their walls cannot always be clearly distinguished, a margin of error is to be considered on the estimated diameter values. Standard Doppler images were calculated to identify the different heartbeat cycles from the phase variations in arteries over the full acquisition. For each cardiac cycle, the subset of intensity and phase B-scans were extracted from the entire sequence for the subsequent analysis. In the case of a Doppler angle close to 90°, the velocity could not be retrieved from the standard Doppler (see Section 4).

#### 2.3.2 Processing of the phase data over time

In order to analyze RNF tissue changes over individual or several consecutive cardiac cycles, i.e. motion in the range of seconds, we used the two TPE methods, M1 and M2, introduced in section 2.1. For the evaluation of a single heartbeat sequence or a sequence of consecutive heartbeat cycles, several post-processing steps were followed as described below for M1:

*Step 1.* The first frame (phase and intensity data) of the heartbeat cycle was selected as reference for all subsequent steps. This corresponds to the beginning of the systolic phase. In the case of consecutive cardiac cycles, the reference frame is the first frame of the first heartbeat cycle for the processing of the entire sequence. The influence of the selection of the reference frame on the evaluated data is outlined in Section 4.

*Step 2.* Registration was then performed: the shifts in x and z direction for each scan compared to the reference scan were obtained using the intensity data and applied to the phase data.

*Step 3.* Preliminary phase difference tomograms between the reference phase B-scan and each subsequent phase B-scan (at each recorded instant of the heartbeat cycle) were calculated.

*Step 4.* Bulk motion correction at each A-scan position was calculated on each preliminary phase difference scan to remove the remaining small amplitude motion. Two methods were implemented: either taking the mean phase difference value over the entire A-scan depth or taking the mean value over the pixels at the IS/OS junction only (as marked between the red dotted lines in Fig. 2(b)).

*Step 5.* The final bulk motion corrected phase difference tomograms between the reference phase B-scan and each subsequent phase B-scan were calculated.

For M2, the same processing steps are followed using a moving reference, i.e. taking the phase difference tomograms between adjacent B-scans, instead of a fixed reference (here the first frame of a heartbeat cycle). The sequence of deformation B-scans over time is obtained by temporal integration of the individual phase difference scans starting with the reference scan followed by conversion to tissue deformation in µm using Eq. (1).

**Fig. 4.**
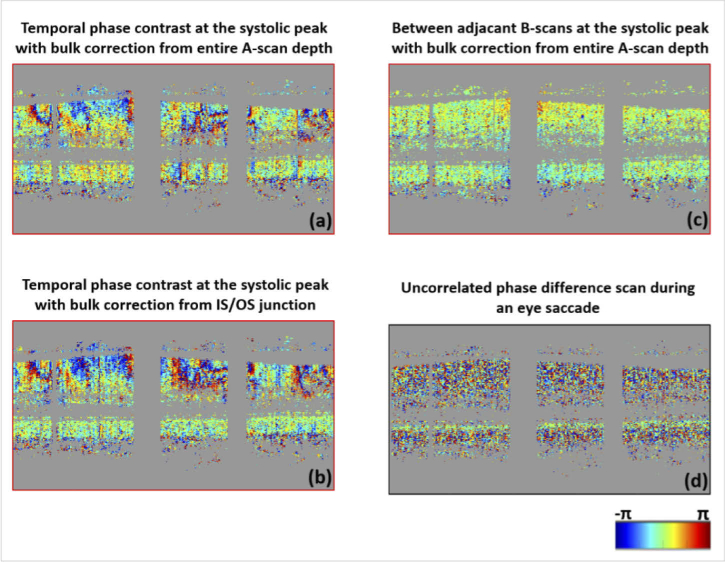
Example of a phase difference scan at the systolic peak for S1 obtained with the M1 with the bulk correction using the entire A-scan depth (a) and only the pixels at the IS/OS junction (b). The scans correspond to the systolic peak marked in red in Fig. 3 (c). Large phase changes in the entire scan can be seen, with greater contrast in (b). For comparison, a phase difference scan between adjacent B-scans at the systolic peak is given in (c) (corresponds to one frame of M2), where only a slight increase in the values is seen around the vessels. Eye saccades result in de-correlated scans as shown in (d), corresponding to the saccade marked in black in Fig. 3 (b) and (c).

Examples of the phase difference tomograms (obtained using M1) at the systolic peak of a cardiac cycle using each of the two bulk correction methods are given in Fig. 4(a) and (b). These phase difference tomograms correspond to the difference between the reference frame and the 15^th^ B-scan after the reference frame, corresponding to a time lag of Δτ = 0.14 s. For comparison, a phase difference tomogram between two adjacent B-scans (Δτ ⋍ 9 ms, corresponding to the procedure of OCTA imaging) is shown in Fig. 4(c). While Figs. 4(a), and (b) indicate absolute movements (with respect to the reference frame), the color coding of Fig. 4(c) is proportional to the speed of tissue motion. Compared to the latter case, large changes with several wrappings are seen in the RNF tissue surrounding the vessels over the entire scan taken with the long Δτ values. The phase difference data corresponding to the A-scans containing the large vessels are unreliable because of the low intensity in these parts of the images that prevent a reliable bulk phase correction. Therefore, these areas are shown in gray. By using only the data from the IS/OS junction for bulk motion correction, higher contrast is maintained in the RNFL phase difference pattern while the phase difference values at the IS/OS are about zero (Fig. 4(b)). This is expected as taking the entire depth range for bulk correction (Fig. 4(a)) compensates some of the motion of the RNFL itself. The IS/OS based bulk motion correction was used for the remainder of this study, assuming that the IS/OS phase information is stable over such period of time.

Despite using the retinal tracker during acquisition and registration in the post-processing steps, eye saccades may be seen in some sequences. Such frames can easily be recognized and isolated as the phase correlation is lost and no pattern can be extracted from the phase difference scans (only the IS/OS green band is recognizable) as shown in Fig. 4(d).

#### 2.3.3 Evaluation of the cardiac cycles sequences

After demonstration of the tissue motion in the individual consecutive phase difference scans, we aim to analyze and quantify the difference in RNF tissue deformation near veins and arteries over a full cardiac cycle. Such evaluation was done using RNFL deformation maps calculated in the following way for M1:

*Step 1.* The individual phase difference scans were flattened after segmentation of the upper boundary of the RNFL on the averaged intensity image.

*Step 2.* For each flattened phase difference tomogram, a band of five pixels along an A-scan (pixels i-2, i-1, i, i+1, i+2) at a given RNFL depth i was extracted and the data of the five pixels were averaged in complex space, similarly as done using the VM vector in OCE [56]. The data were additionally smoothened by averaging over the neighboring pixels (j-1, j+1) at a given A-scan j and over two consecutive bands in time.

*Step 3.* A wrapped temporal phase evolution OCT map (wrapped TPE map), i.e. an M-scan of phase evolution as a function of position along B-scan trace vs. time, of the smoothened phase difference corresponding to depth position i in the RNFL tissue, was calculated and plotted, covering one or several cardiac cycles.

*Step 4.* The corresponding deformation map (unwrapped TPE map) was obtained after unwrapping of the data, using the MATLAB unwrap function. The unwrapped map was smoothened by a Gaussian filter and converted to tissue deformation in µm using the relation below:

(1) $$\Delta \text{u}=\frac{\Delta \varphi \;*\;\lambda_{0}}{4\pi n}$$

with Δφ the phase difference value at each pixel, λ_0_ the center wavelength of the illumination light and n the refractive index of the medium (1.38 in this study).

*Step 5.* Representative plots of the RNF tissue deformation were finally extracted close to each artery or vein by selecting a reference profile (tissue deformation over time for a given A-scan position) at the largest deformation and taking the mean of the 5 most correlated adjacent profiles to that reference profile.

For M2, similar steps were performed for the phase difference data between adjacent B-scans. The final deformation map is retrieved by temporal integration instead of unwrapping.

## 3. Results

### 3.1 Visualization of RNFL deformation

**Fig. 5.**
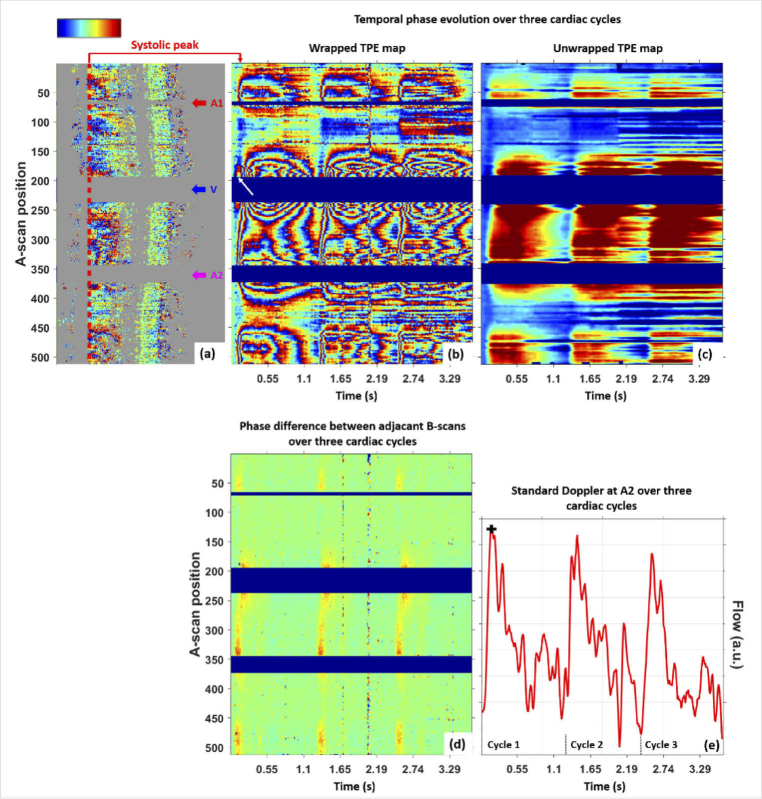
Tissue motion analysis of RNFL over time. Wrapped (b) and unwrapped (c) RNFL TPE map (M-scan of phase/deformation evolution as a function of position along B-scan trace vs. time) at the location marked by red dotted line in (a) over three cardiac cycles for S1 are obtained using M1. The wrapping pattern repeats itself over the cardiac cycles in (b) corresponding to the deformations seen in (c). A similar TPE map as given in (c) is obtained after integration of the map (d) as done in M2 (not shown here). (e) Standard Doppler analysis extracted from artery A2 (A-scan 360) over the same temporal range. Colorbar: -π to π for (a), (b) and (d), <-0.25 µm to > 0.75 µm for (c).

An example of a RNFL TPE map over three cardiac cycles, i.e. about 3.5 s, for S1 is given in Fig. 5 (corresponding to the intensity sequence of Fig. 3(b)). The maps were obtained at the RNFL band marked in red in (a) using M1. Large phase changes, including several phase wraps, are observed in the wrapped TPE map (b), indicating axial tissue motions beyond half a wavelength. In this map, the first frame of the first cardiac cycle is taken as reference for the calculation of the phase difference images to each of the subsequent scans. The wrapping pattern repeats itself over the three cardiac cycles. The TPE map given in (c) represents the tissue deformation after unwrapping and smoothing of the data displayed in (b). In the image displaying the phase difference between consecutive B-scans (d), changes in the RNFL are only seen close to the vessels during the systolic phase for each of the three heartbeat cycles as this data is related to the velocity of tissue. An integration of the map (d) along the temporal axis provides a comparable deformation map as given in (c). A video with the sequences of the individual intensity, phase difference scans (using M1) and deformation scans (using M2) over one cardiac cycle is provided in the supplementary material (Visualization 1). From a visual point of view, the changes in tissue expansion from frame to frame are better emphasized in the phase difference scans (using M1) while the depth extension of the deformation is better seen on the integrated deformation scans (using M2).

The acquired trace (B-scan) of Fig. 5 is crossing two arteries at A-scan locations 70 and 350 and a vein at location 200. While expansion starts in the RNF tissue around the arteries a few frames after the reference frame, a slight shrinkage is first seen on the vein pattern before expansion (marked by a white arrow in Fig. 5(b)). This may be explained by the fact that the tissue did not reach its fully contracted state at the time the reference frame was taken, i.e., there is a lag between arterial and venous pulsations. The behavior of the tissue deformation is further detailed in the next section 3.2, comparing the results for several subjects. An interesting pattern can be observed between the vein and the larger artery, i.e. between the A-scan positions 250 and 340. This pattern could be potentially caused by interference of mechanical tissue waves. The wrapped TPE and intensity maps, as a function of depth beneath the RNFL surface, recorded over one cardiac cycle, are presented in supplementary material (Visualization 2).

**Fig. 6.**
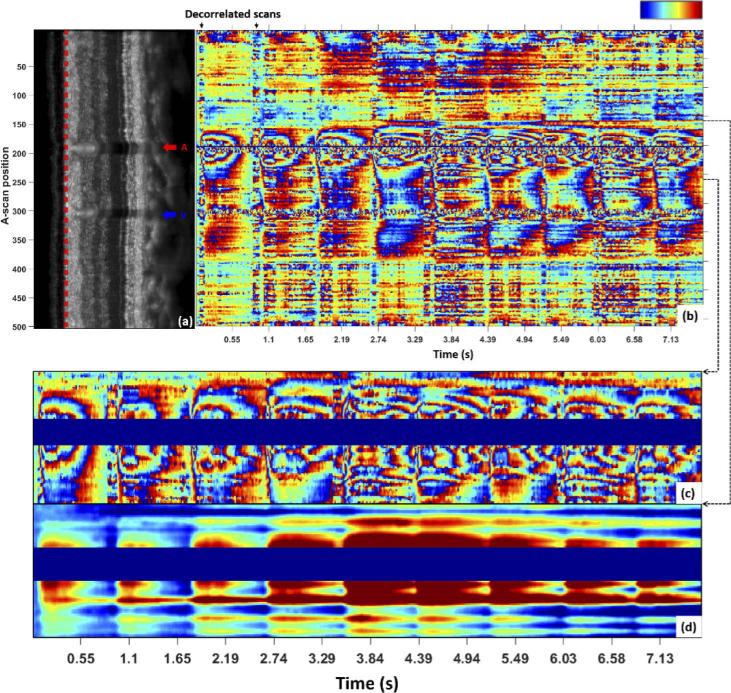
RNFL TPE map recorded over an extended time of ∼ 8 s (b) at the top of the RNFL of S2 at position marked by red-dashed line in (a). Good phase stability is observed over most of the recording period, with exception of few de-correlated scans such as the ones marked by arrows. The reference scan was selected at the beginning of the first cardiac cycle for the entire sequence of nine cycles. A zoomed TPE map centered on the artery A is given in (c) with its corresponding deformation map in (d) obtained using M2. The variations in the extent of the tissue deformation over the different cycles is especially visible on the deformation map. Colorbar: -π to π for (b) and (c), <-0.25 µm to > 0.75 µm for (d).

Figure 6 presents the RNFL deformation of another healthy subject over nine heartbeat cycles, i.e. almost 8 s, with the reference frame taken at the first scan of the first cycle. This example demonstrates the good phase stability over the acquisition sequence, which is ensured by the retinal tracker during acquisition. Despite some de-correlated scans, as seen for example at the beginning of the first cycle and probably due to an eye saccade, the phase stability is clearly retrieved in the next scans. This trace crosses both an artery and a vein, as observed in the center part of the averaged intensity B-scan. An additional small vessel can also be distinguished at the edge of the trace (at ∼ A-scan no. 6), causing additional RNF tissue deformation. A similar repetitive wrapping pattern under pulsation as in Fig. 5(b) is obtained (Fig. 6(b)). Less wrapping can however be observed, probably due to the smaller size of these vessels. The center artery and vein of Fig. 6 have diameters of about 67 µm and 58 µm, respectively, while the artery and the vein of Fig. 5 have diameters of about 104 µm and 145 µm, respectively. The amplitude of the deformation also seems to vary depending on the heartbeat cycle. In Fig. 6, the tissue returns to its initial state after each of the two first cycles. However, larger variations in the deformation pattern can be observed between the third and the seventh cycles, probably due to higher perfusion. The tissue seems to expand with the beginning of the next systolic phase before having time to first reach its initial state again. This phenomenon is clearly seen in the zoomed TPE maps on the center artery given in (c) and (d). The deformation map (d) was obtained using M2.

### 3.2 Investigation of deformation properties

The RNF tissue deformation near arteries and veins under pulsation are compared for the three healthy subjects of Fig. 2 (traces marked in red dotted lines on the LSLO images). For each subject, the three best-quality heartbeat cycles obtained in the 10 s recording time (higher coefficient of correlation over the sequence duration and lower amount of eye saccades) were considered in this analysis. The reference frame was taken at the beginning of each cardiac cycle, considering the three repetitions separately.

**Fig. 7.**
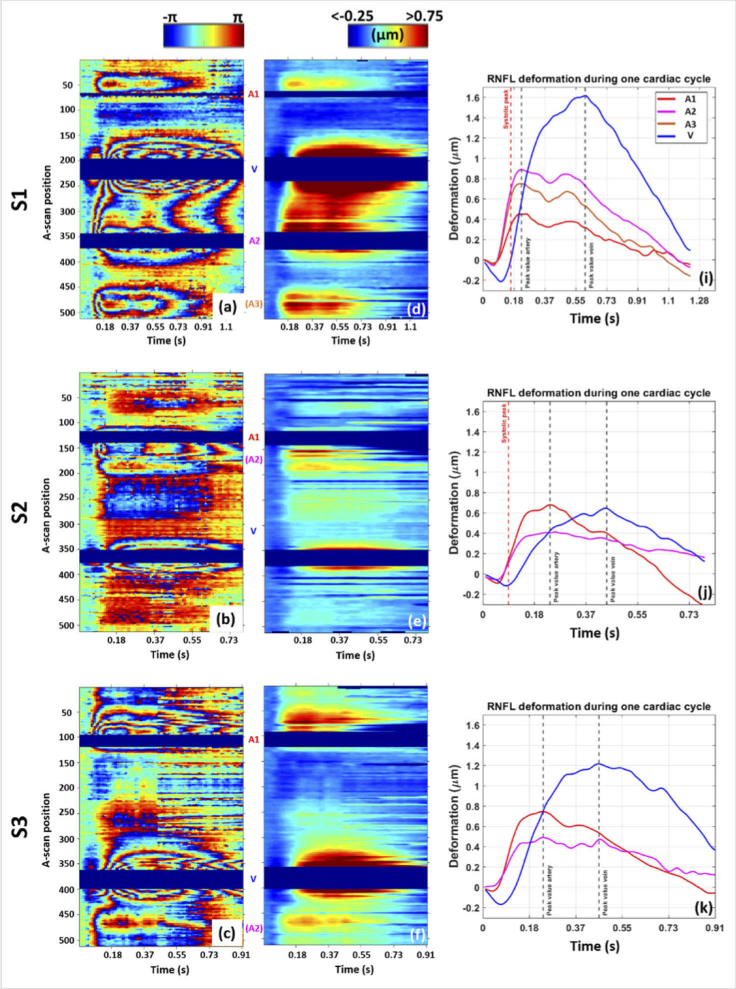
Comparison of the RNF tissue deformation over one cardiac cycle for the three study subjects evaluated with the M1. For S1, S2 and S3 respectively: (a), (b) and (c) are the RNFL wrapped TPE maps at the top boundary of the RNFL, (d), (e) and (f) are the corresponding RNFL TPE deformation maps after unwrapping, (i), (j) and (k) are the deformation profiles extracted from (d), (e), and (f), respectively, close to each artery and vein. Similar tissue deformation patterns are observed in the three subjects. The expansion of the veins is delayed compared to the one of the arteries.

Figure 7 gives examples of the wrapped temporal phase evolution maps and unwrapped deformation maps of one cycle for each volunteer using M1. Despite the difference in maximum deformation observed, similar patterns are seen for all subjects. The expansion in the tissue surrounding the arteries is starting earlier during the systolic phase as compared to the expansion surrounding the veins. The plots given in Figs. 7(i), (j), and (k) confirm this behavior. A double hump pattern can be recognized for the tissue expansion near the arteries, probably corresponding to the peak of the systolic phase and the dicrotic notch. Only a single, broad expansion peak is observed around the veins, reaching significantly higher deformation as compared to the arteries for S1 and S3.

The results obtained in the three repetitive cycles are listed in Table 1 providing vessel diameters and RNFL tissue deformation parameters relative to the reference frame at beginning of the heartbeat cycle. A maximum expansion of 1.58 ± 0.10 µm was obtained at the vein of S1. The average precision for a single pixel (standard deviation) of tissue deformation measurements is ∼ 30 nm. It is obtained from the analysis of the noise values in an area without pulsation on the wrapped TPE scan of S1. Also temporal measurements (such as the time at which this maximum is reached within a cardiac cycle) shows a good repeatability. From Table 1, we calculated the temporal delays between tissue responses around arteries and veins. Delays of 0.35 s, 0.15 s and 0.27 s (corresponding to about 29%, 19% and 30% of the total time of the cycle) were obtained between the maximum deformation reached at the arteries and the one reached at the veins for S1, S2 and S3, respectively. Delays between the beginning of the tissue expansion at the arteries and at the veins were found to be 0.05 s, 0.04 s and 0.05 s for S1, S2 and S3, respectively. Similarly, delays of 0.08 s, 0.04 s and 0.08 s are calculated for the maximum of the gradient during the tissue expansion (maximum tissue expansion speed (du/dt)_max_).

**Table 1.** Vessel diameters and RNFL tissue deformation parameters (Δu and times relative to the reference frame) in the vicinity of vessels observed in three healthy volunteers. Mean values ± standard deviation for three cardiac cycles. Right before the arriving of the pulse wave, the tissue reaches its maximum contraction Δu_min_. While the pulse wave propagates through the tissue, the maximum tissue expansion speed (du/dt)_max_ is then reached. The tissue expands up to a maximum value Δu_max_ before it starts retracting. NC = not calculable.

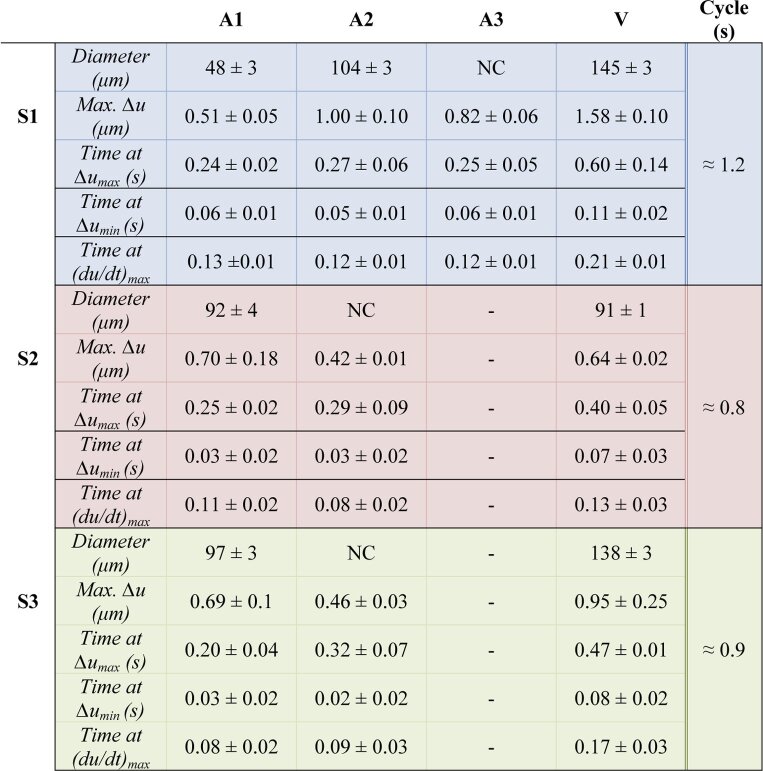

## 4. Discussion

We demonstrated the use of TPE OCT to analyze RNF tissue deformation induced by retinal vessels pulsating with the heartbeat. Using a custom-built SD-OCT system with an integrated retinal tracker, we acquired phase stable measurements over several heartbeat cycles in different healthy subjects. After processing of the TPE data, a reproducible phase pattern was observed in the vicinity of the retinal vessels over the course of the different cardiac cycles. From the unwrapped deformation maps, we could quantify and compare the tissue deformation near arteries and veins with an average precision for a single pixel of about 30 nm. The theoretical value, calculated as introduced in [57], is about 15 nm (based on an average SNR of 10 dB computed from the ratio of the average signal in the RNFL and the average noise).

Probing the biomechanical properties of ocular tissues has been of increasing interest, especially with the development of OCE and its capability to access tissue elastic modulus. Non-invasive OCE measurements are however still challenging in the eye and have essentially been limited to the cornea in human subjects *in-vivo* [24]. Compared to standard OCE which uses an external pulse source, our method relies on the natural cardiac pulsation to investigate tissue deformation, similarly to recent work reported on passive elastography in the cornea in humans [37] and in animal models [36]. While such method does not provide information on the elastic modulus (the force exerted on the tissue is unknown), it gives interesting insights on retinal tissue motion over the range of several seconds. In a further step, strain estimation could be performed based either on the LSM [18], as in heartbeat OCT [36], or the VM [19] and optimized VM [20,21].

Another PhS-OCT method that provides access to motion on a larger time scale was presented by Spahr et al., using a FF-SS-OCT system [38]. This method uses a very fast area camera to record retinal OCT volumes at a volume rate of 2000 volumes/s. From these volumes, 2D en-face images corresponding to two retinal layers (RPE and RNFL) are segmented, and the phase difference between these layers is obtained. From the temporal evolution of these phase differences, tissue deformation over time is derived by temporal integration of phase difference changes. An advantage of this method is the access to tissue deformation over a 2D en face area. The technique is, however, more sensitive to multiple scattered photons and artifacts caused by fast motion within vessels may deteriorate the image quality. Using FF-OCT, the choice of the imaging parameters is also less flexible compared to standard scanning OCT systems, which can be a limiting factor for various measurements. Additionally, scanning systems are already commonly used in clinical applications, while clinical translation of FF systems is still pending.

In order to retrieve motion over the range of seconds, high phase stability is essential during acquisition. Despite the moderate imaging speed of our SD-OCT system (A-scan rate of 70 kHz) compared to recent ultra-fast OCT systems [43], phase stability is ensured by the sub-speckle precision of the integrated retinal tracker [46,52]. In this study, we demonstrated phase stable measurements over a sequence of nine cardiac cycles, corresponding to about 8 s. During eye saccades, the phase correlation may be lost over a few scans but is retrieved in the subsequent scans. While we only acquired sequences of about 10 s (1000 B-scans in total), the phase stability could potentially be preserved over longer time, after adaptation of the acquisition software. In addition to the phase stability of the system, compliance of the imaged subject is essential for such long measurements. Over longer timeframes, rotation and other misalignments during acquisition may deteriorate the phase stability. The method was so far only applied to a few healthy volunteers, with good fixation skills and selecting cross-section traces of about 2 mm length. Imaging of a larger set of healthy subjects and/or patients with reduced fixation skills may require further adaptation of the system. For example, the robustness of the imaging method could be improved by implementing rotation correction on the retinal tracker (the current version only correcting translation).

After the recording of phase stable measurements, an essential post-processing step for the purpose of our analysis is the bulk motion correction. In standard Doppler OCT and OCTA, various methods have been used, as shown for example in [58,59]. In our analysis we tested two different methods: correcting the phase difference scans by the mean phase difference value over the entire depth at each A-scan position or by only considering the mean of the pixels corresponding to the IS/OS junction. We observed that the second method provides higher contrast in the TPE scans, as the first method is incorrectly compensating some of the RNF tissue motion. We chose to use the bulk motion correction taking only the IS/OS data, under the assumption that no or low motion will happen in this layer in the timeframe of our analysis (a few seconds) as compared to the larger RNFL motion induced by vessel pulsation. All quantitative measurements were performed on single and separately analyzed heartbeat cycles taking the reference frame at the beginning of each cycle. For a quantitative evaluation over longer time, for example taking the reference frame at the beginning of a first cycle and analyzing several subsequent cycles as in Fig. 6, the impact of slower phase changes at the photoreceptor layer will need to be further investigated.

Another important step of the data processing is the choice of the reference frame for the TPE analysis. As our study focuses on motion induced by vessel pulsation, we chose to take the reference scan at the beginning of a cardiac cycle. The choice of the reference frame will influence both, the computation of each phase difference tomogram using M1 and the temporal integration using M2. In this work, we determined the reference frame from the standard Doppler OCT data derived at the artery location. However, in the case of a Doppler angle close to 90° or when imaging smaller arteries, the Doppler signal may be noisier or not accessible. In these cases (such as S3 in this study), the beginning of the heartbeat cycle was determined by manually shifting the reference frame until obtaining a phase pattern similar to those usually observed near the retinal vessels (cf., e.g., Fig. 5(b)). Four other healthy volunteers were imaged additionally to the three study subjects, as we tested different acquisition parameters, and similar phase patterns at the arteries and veins have been observed in each case.

**Fig. 8.**
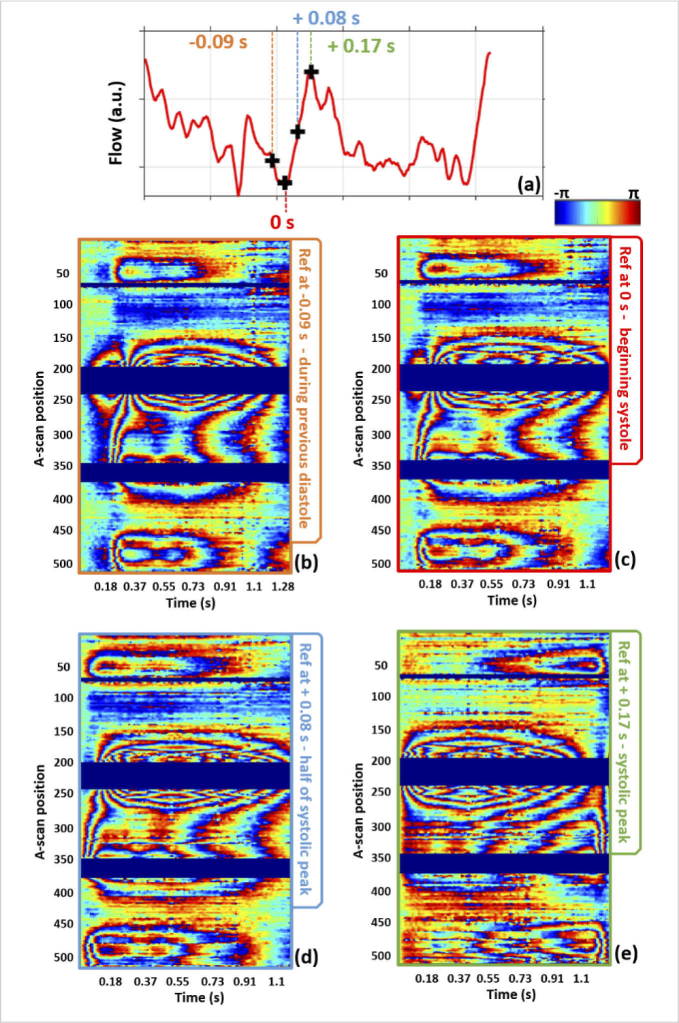
Influence of shifting the reference frame on the measured phase difference. The TPE maps of S1 over the time frame of one cardiac cycle were calculated setting the reference frame at (b) the end of the previous diastolic phase, (c) the beginning of the systolic phase, (d) half of the systolic peak, (e) the systolic peak. The maps (b)-(d) are plotted until the end of the cardiac cycle and the map (e) until the systolic peak of the next cardiac cycle. Time points of the selected reference frames are marked in the standard Doppler OCT plot (derived at the position of the artery at A-scan 360) in (a).

To further evaluate the influence of the reference scan on the results and to improve the interpretation of the data, we investigated the use of different reference frames for the B-scan trace of S1 as shown in Fig. 8. Additionally to the usual wrapped TPE map (c) with the reference frame at the beginning of the systolic phase, we reprocessed the data after choosing different reference frames: during the previous diastolic phase (0.09 s before the start of the systolic phase) (b), at half of the systolic peak (d) and at the systolic peak (e). When setting the reference frame at the end of the previous diastolic phase, we can see a larger tissue shrinkage with several phase wraps near the vein as well as a slight shrinkage near the arteries before expansion. When setting the reference frame at the beginning of the systolic phase and then at half of the systolic peak, this shrinkage is not seen any more close to the arteries. In these cases, the expansion of the arteries started after 0.1 s with the reference frame at the beginning of the systolic phase and right away with the reference frame at half of the systolic peak. Similarly, only a reduced tissue retraction is seen close to the vein before expansion on these images. Taking the reference frame at the systolic peak, the tissue expands directly near the vein while the usual double hump wrapping pattern is lost near the arteries. Only a slight tissue expansion can be distinguished before the RNF tissue shrinks again at the top and bottom arteries. Some more distorted phase wrapping patterns can still be seen near the middle artery, possibly due to the pattern of tissue wave interference between this artery and the vein. The quantitative analysis of tissue deformation is thus greatly dependent on the choice of the reference frame.

Data evaluation method M1 seems especially useful for analyzing subtle details of tissue deformation, visualized by phase difference contours that immediately show patterns of sub-wavelength deformation. To quantitatively analyze larger tissue deformation (larger than half the wavelength of the OCT sampling beam), phase unwrapping is used to convert the phase data into absolute distances. We used here the built-in MATLAB unwrapping function. The RNF tissue deformation measured in this study showed peak values up to about 1.6 µm, which is in agreement with literature data [38]. In the case of noisier data (as it was the case for some of the data of the additionally imaged subjects), the built-in algorithm failed to correctly unwrap the phase difference information of M1. The development of a custom algorithm might be beneficial that is less sensitive to the varying quality of datasets and that excludes unwrapping errors, for example caused by eye saccades*.* As only small displacements are induced between consecutive B-scans, unwrapping and therefore unwrapping errors are avoided using M2, which seems therefore especially suited for quantification of larger tissue deformation, as, e.g., for visualization of the depth extension of tissue deformation as shown in Visualization 1. In the case of higher rate motions, as for example seen in OCE [60], an additional unwrapping step may however be required. Eye saccades may nonetheless affect the performance of the temporal integration in M2. Especially in areas where only little motion occurs, inaccuracies in the phase difference values may have a stronger influence on the cumulated data. Optimization of the processing steps will permit a larger quantitative analysis and be particularly important for future work in order to evaluate patient data, which may be of lower quality. Implementation of the extended Knox-Thompson algorithm for the computation of the phase difference data, may additionally improve the overall quality of the processed data [50].

Our study showed that TPE OCT is able to provide measurement of tissue deformation with resolution in the 10 nm range over extended times. Possible applications comprise, e.g., the investigation of pulse wave propagation, the temporal analysis of tissue deformation, as well as time lags between motions induced by arterial and venous pulsations, under normal physiologic conditions, but also in various ocular disorders that are known to be associated with altered blood circulation, like glaucoma, diabetic retinopathy, or retinal vein occlusions. Moreover, our method might also be useful for analyzing cardiovascular diseases, e.g. by looking for changes in tissue deformation patterns near vessels that are affected by various types of plaques. On a longer term (and more speculative), by using the retina as a “window into the brain”, our method might be useful to identify patients at risk of brain damage from vascular complications. Other possible applications could comprise, e.g., the analysis of perfusion changes under visual stimulus, which could complement other recent optophysiologic and opto-retinographic studies [61–63] and thereby support various physiologic studies in the field of vision research.

## 5. Conclusion

In this work, we investigated the TPE method for measuring deformations in retinal tissue over the range of seconds. We evaluated this deformation in the RNFL in the vicinity of retinal vessels under cardiac pulsation in healthy human subjects. The integrated retinal tracker of the OCT system used for imaging, providing sub-speckle precision, ensured phase stability during acquisition. Reproducible results with a precision of about 30 nm were obtained and differences between deformation induced by veins and arteries of different caliber could be evaluated. In future work, TPE analysis of tissue deformation may provide valuable new information not only for the monitoring of ocular disorders but also other diseases affecting the blood circulation such as cardiovascular ones.

## Data Availability

Data underlying the results presented in this paper are not publicly available at this time but may be obtained from the authors upon reasonable request.

## References

[1] Patton N. Aslam T. MacGillivray T. Pattie A. Deary I. J. Dhillon B., “Retinal vascular image analysis as a potential screening tool for cerebrovascular disease: a rationale based on homology between cerebral and retinal microvasculatures,” J. Anat. 206(4), 319–348 (2005). 10.1111/j.1469-7580.2005.00395.x 15817102 PMC1571489

[2] McClintic B. R. McClintic J. I. Bisognano J. D. Block R. C., “The relationship between retinal microvascular abnormalities and coronary heart disease: a review,” The American Journal of Medicine 123(4), 374.e1 (2010). 10.1016/j.amjmed.2009.05.030 PMC2922900 20362758

[3] Chen J. J., “Optical coherence tomography and neuro-ophthalmology,” Journal of Neuro-Ophthalmology 38(1), 1–3 (2018). 10.1097/WNO.0000000000000628 28266953

[4] Rim T. H. Teo A. W. J. Yang H. H. S. Cheung C. Y. Wong T. Y., “Retinal vascular signs and cerebrovascular diseases,” Journal of Neuro-Ophthalmology 40(1), 44–59 (2020). 10.1097/WNO.0000000000000888 31977663

[5] Cerveró A. Casado A. Riancho J., “Retinal changes in amyotrophic lateral sclerosis: looking at the disease through a new window,” J. Neurol. 268(6), 2083–2089 (2021). 10.1007/s00415-019-09654-w 31792674

[6] Kashani A. H. Chen C. L. Gahm J. K. Zheng F. Richter G. M. Rosenfeld P. J. Shi Y. Wang R. K., “Optical coherence tomography angiography: a comprehensive review of current methods and clinical applications,” Prog Retin Eye Res 60, 66–100 (2017). 10.1016/j.preteyeres.2017.07.002 28760677 PMC5600872

[7] Leitgeb R. A. Werkmeister R. M. Blatter C. Schmetterer L., “Doppler optical coherence tomography,” Prog. Retinal Eye Res. 41, 26–43 (2014). 10.1016/j.preteyeres.2014.03.004 PMC4073226 24704352

[8] Jonnal R. S. Kocaoglu O. P. Wang Q. Lee S. Miller D. T., “Phase-sensitive imaging of the outer retina using optical coherence tomography and adaptive optics,” Biomed. Opt. Express 3(1), 104–124 (2012). 10.1364/BOE.3.000104 22254172 PMC3255329

[9] Pfäffle C. Spahr H. Kutzner L. Burhan S. Hilge F. Miura Y. Hüttmann G. Hillmann D., “Simultaneous functional imaging of neuronal and photoreceptor layers in living human retina,” Opt. Lett. 44(23), 5671–5674 (2019). 10.1364/OL.44.005671 31774751

[10] Wang R. K. Nuttall A. L., “Phase-sensitive optical coherence tomography imaging of the tissue motion within the organ of Corti at a subnanometer scale: a preliminary study,” J. Biomed. Opt. 15(5), 056005 (2010). 10.1117/1.3486543 21054099 PMC2948044

[11] Li C. Guan G. Huang Z. Wang R., “Quantitative elastography of skin and skin lesion using phase-sensitive OCT (PhS-OCT) and surface wave method,” >SPIE BiOS (SPIE, 2012), Vol. 8213.

[12] Hamidi A. Bayhaqi Y. Canbaz F. Navarini A. Cattin P. Zam A., “Imaging photothermal-induced expansion of bone during laser osteotomy by phase-sensitive OCT: preliminary results,” SPIE Photonics Europe (SPIE, 2020), Vol. 11359.

[13] Kennedy B. F. Kennedy K. M. Sampson D. D., “Optical coherence elastography,” Optics & Photonics News 26(4), 32–39 (2015). 10.1364/OPN.26.4.000032

[14] Larin K. V. Sampson D. D., “Optical coherence elastography: OCT at work in tissue biomechanics [Invited],” Biomed. Opt. Express 8(2), 1172–1202 (2017). 10.1364/BOE.8.001172 28271011 PMC5330567

[15] Wang R. K. Ma Z. Kirkpatrick S. J., “Tissue Doppler optical coherence elastography for real time strain rate and strain mapping of soft tissue,” Appl. Phys. Lett. 89(14), 144103 (2006). 10.1063/1.2357854

[16] Wang R. K. Kirkpatrick S. Hinds M., “Phase-sensitive optical coherence elastography for mapping tissue microstrains in real time,” Appl. Phys. Lett. 90(16), 164105 (2007). 10.1063/1.2724920

[17] Li Y. Moon S. Chen J. J. Zhu Z. Chen Z., “Ultrahigh-sensitive optical coherence elastography,” Light: Sci. Appl. 9(1), 58 (2020). 10.1038/s41377-020-0297-9 32337022 PMC7154028

[18] Kennedy B. F. Koh S. H. McLaughlin R. A. Kennedy K. M. Munro P. R. T. Sampson D. D., “Strain estimation in phase-sensitive optical coherence elastography,” Biomed. Opt. Express 3(8), 1865–1879 (2012). 10.1364/BOE.3.001865 22876350 PMC3409705

[19] Zaitsev V. Matveyev A. Matveev L. Gelikonov G. Sovetsky A. Vitkin A., “Optimized phase gradient measurements and phase-amplitude interplay in optical coherence elastography,” J. Biomed. Opt. 21(11), 116005 (2016). 10.1117/1.JBO.21.11.116005 27824215

[20] Zaitsev V. Y. Matveev L. A. Matveyev A. L. Sovetsky A. A. Shabanov D. V. Ksenofontov S. Y. Gelikonov G. V. Baum O. I. Omelchenko A. I. Yuzhakov A. V. Sobol E. N., “Optimization of phase-resolved optical coherence elastography for highly-sensitive monitoring of slow-rate strains,” Laser Phys. Lett. 16(6), 065601 (2019). 10.1088/1612-202X/ab183c

[21] Bai Y. Cai S. Xie S. Dong B., “Adaptive incremental method for strain estimation in phase-sensitive optical coherence elastography,” Opt. Express 29(16), 25327–25336 (2021). 10.1364/OE.433245 34614865

[22] Hillmann D. Spahr H. Pfäffle C. Sudkamp H. Franke G. Hüttmann G., “In vivo optical imaging of physiological responses to photostimulation in human photoreceptors,” Proc. Natl. Acad. Sci. 113(46), 13138–13143 (2016). 10.1073/pnas.1606428113 27729536 PMC5135337

[23] Guan G. Reif R. Wang R. Huang Z., “Depth profiling of photothermal compound concentrations using phase sensitive optical coherence tomography,” J. Biomed. Opt. 16(12), 126003 (2011). 10.1117/1.3659211 22191920 PMC3247934

[24] Kirby M. A. Pelivanov I. Song S. Ambrozinski Ł. Yoon S. J. Gao L. Li D. Shen T. T. Wang R. K. O’Donnell M., “Optical coherence elastography in ophthalmology,” J. Biomed. Opt. 22(12), 1–28 (2017). 10.1117/1.JBO.22.12.121720 PMC5745712 29275544

[25] Ramier A. Tavakol B. Yun S.-H., “Measuring mechanical wave speed, dispersion, and viscoelastic modulus of the cornea using optical coherence elastography,” Opt. Express 27(12), 16635–16649 (2019). 10.1364/OE.27.016635 31252887 PMC6825608

[26] Zvietcovich F. Nair A. Singh M. Aglyamov S. R. Twa M. D. Larin K. V., “Dynamic optical coherence elastography of the anterior eye: understanding the biomechanics of the limbus,” Invest. Ophthalmol. Visual Sci. 61(13), 7 (2020). 10.1167/iovs.61.13.7 PMC7645208 33141893

[27] Lal C. Alexandrov S. Rani S. Zhou Y. Ritter T. Leahy M., “Nanosensitive optical coherence tomography to assess wound healing within the cornea,” Biomed. Opt. Express 11(7), 3407–3422 (2020). 10.1364/BOE.389342 33014541 PMC7510923

[28] De Stefano V. S. Ford M. R. Seven I. Dupps W. J. Jr., “Live human assessment of depth-dependent corneal displacements with swept-source optical coherence elastography,” PLoS One 13(12), e0209480 (2018). 10.1371/journal.pone.0209480 30592752 PMC6310362

[29] Jin Z. Chen S. Dai Y. Bao C. Ye S. Zhou Y. Wang Y. Huang S. Wang Y. Shen M. Zhu D. Lu F., “In vivo noninvasive measurement of spatially resolved corneal elasticity in human eyes using Lamb wave optical coherence elastography,” J. Biophotonics 13, e202000104 (2020). 10.1002/jbio.202000104 32368840

[30] Lan G. Aglyamov S. R. Larin K. V. Twa M. D., “In vivo human corneal shear-wave optical coherence elastography,” Optom. Vis. Sci. 98(1), 58–63 (2021). 10.1097/OPX.0000000000001633 33394932 PMC7774819

[31] Ramier A. Eltony A. M. Chen Y. Clouser F. Birkenfeld J. S. Watts A. Yun S.-H., “In vivo measurement of shear modulus of the human cornea using optical coherence elastography,” Sci. Rep. 10(1), 17366 (2020). 10.1038/s41598-020-74383-4 33060714 PMC7567833

[32] Song S. Le N. M. Huang Z. Shen T. Wang R. K., “Quantitative shear-wave optical coherence elastography with a programmable phased array ultrasound as the wave source,” Opt. Lett. 40(21), 5007–5010 (2015). 10.1364/OL.40.005007 26512505

[33] Qu Y. He Y. Zhang Y. Ma T. Zhu J. Miao Y. Dai C. Humayun M. Zhou Q. Chen Z., “Quantified elasticity mapping of retinal layers using synchronized acoustic radiation force optical coherence elastography,” Biomed. Opt. Express 9(9), 4054–4063 (2018). 10.1364/BOE.9.004054 30615733 PMC6157789

[34] Qu Y. He Y. Saidi A. Xin Y. Zhou Y. Zhu J. Ma T. Silverman R. H. Minckler D. S. Zhou Q. Chen Z., “In vivo elasticity mapping of posterior ocular layers using acoustic radiation force optical coherence elastography,” Invest. Ophthalmol. Visual Sci. 59(1), 455–461 (2018). 10.1167/iovs.17-22971 29368002 PMC5783626

[35] Nguyen T.-M. Zorgani A. Lescanne M. Boccara C. Fink M. Catheline S., “Diffuse shear wave imaging: toward passive elastography using low-frame rate spectral-domain optical coherence tomography,” J. Biomed. Opt. 21(12), 126013 (2016). 10.1117/1.JBO.21.12.126013 27999863

[36] Nair A. Singh M. Aglyamov S. Larin K. V., “Heartbeat optical coherence elastography: corneal biomechanics in vivo,” J. Biomed. Opt. 26(02), 020502 (2021). 10.1117/1.JBO.26.2.020502 PMC7901857 33624461

[37] Kwok S. Clayson K. Hazen N. Pan X. Ma Y. Hendershot A. J. Liu J., “Heartbeat-induced corneal axial displacement and strain measured by high frequency ultrasound elastography in human volunteers,” Trans. Vis. Sci. Tech. 9(13), 33 (2020). 10.1167/tvst.9.13.33 PMC7757631 33384887

[38] Spahr H. Hillmann D. Hain C. Pfäffle C. Sudkamp H. Franke G. Hüttmann G., “Imaging pulse wave propagation in human retinal vessels using full-field swept-source optical coherence tomography,” Opt. Lett. 40(20), 4771–4774 (2015). 10.1364/OL.40.004771 26469616

[39] Xin C. Song S. Johnstone M. Wang N. Wang R. K., “Quantification of pulse-dependent trabecular meshwork motion in normal humans using phase-sensitive OCT,” Invest. Ophthalmol. Visual Sci. 59(8), 3675–3681 (2018). 10.1167/iovs.17-23579 30029254 PMC6054426

[40] Gao K. Song S. Johnstone M. A. Zhang Q. Xu J. Zhang X. Wang R. K. Wen J. C., “Reduced pulsatile trabecular meshwork motion in eyes with primary open angle glaucoma using phase-sensitive optical coherence tomography,” Invest. Ophthalmol. Visual Sci. 61(14), 21 (2020). 10.1167/iovs.61.14.21 PMC7745620 33326017

[41] O’Hara K. Schmoll T. Vass C. Leitgeb R., “Measuring pulse-induced natural relative motions within human ocular tissue in vivo using phase-sensitive optical coherence tomography,” J. Biomed. Opt. 18(12), 121506 (2013). 10.1117/1.JBO.18.12.121506 24194123

[42] Moon S. Chen Z., “Phase-stability optimization of swept-source optical coherence tomography,” Biomed. Opt. Express 9(11), 5280–5295 (2018). 10.1364/BOE.9.005280 30460128 PMC6238911

[43] Klein T. Huber R., “High-speed OCT light sources and systems [Invited],” Biomed. Opt. Express 8(2), 828–859 (2017). 10.1364/BOE.8.000828 28270988 PMC5330584

[44] Lan G. Singh M. Larin K. V. Twa M. D., “Common-path phase-sensitive optical coherence tomography provides enhanced phase stability and detection sensitivity for dynamic elastography,” Biomed. Opt. Express 8(11), 5253–5266 (2017). 10.1364/BOE.8.005253 29188118 PMC5695968

[45] Braaf B. Vienola K. V. Sheehy C. K. Yang Q. Vermeer K. A. Tiruveedhula P. Arathorn D. W. Roorda A. de Boer J. F., “Real-time eye motion correction in phase-resolved OCT angiography with tracking SLO,” Biomed. Opt. Express 4(1), 51–65 (2013). 10.1364/BOE.4.000051 23304647 PMC3539196

[46] Schwarzhans F. Desissaire S. Steiner S. Pircher M. Hitzenberger C. K. Resch H. Vass C. Fischer G., “Generating large field of view en-face projection images from intra-acquisition motion compensated volumetric optical coherence tomography data,” Biomed. Opt. Express 11(12), 6881–6904 (2020). 10.1364/BOE.404738 33408968 PMC7747913

[47] Bartuzel M. M. Wróbel K. Tamborski S. Meina M. Nowakowski M. Dalasiński K. Szkulmowska A. Szkulmowski M., “High-resolution, ultrafast, wide-field retinal eye-tracking for enhanced quantification of fixational and saccadic motion,” Biomed. Opt. Express 11(6), 3164–3180 (2020). 10.1364/BOE.392849 32637248 PMC7316009

[48] Chen C.-L. Wang R. K., “Optical coherence tomography based angiography [Invited],” Biomed. Opt. Express 8(2), 1056–1082 (2017). 10.1364/BOE.8.001056 28271003 PMC5330554

[49] Knox K. T. Thompson B. J., “Recovery of images from atmospherically degraded short-exposure photographs,” ApJ 193, L45 (1974). 10.1086/181627

[50] Spahr H. Pfäffle C. Burhan S. Kutzner L. Hilge F. Hüttmann G. Hillmann D., “Phase-sensitive interferometry of decorrelated speckle patterns,” Sci. Rep. 9(1), 11748 (2019). 10.1038/s41598-019-47979-8 31409819 PMC6692410

[51] Grulkowski I. Gorczynska I. Szkulmowski M. Szlag D. Szkulmowska A. Leitgeb R. A. Kowalczyk A. Wojtkowski M., “Scanning protocols dedicated to smart velocity ranging in spectral OCT,” Opt. Express 17(26), 23736–23754 (2009). 10.1364/OE.17.023736 20052085

[52] Sugita M. Zotter S. Pircher M. Makihira T. Saito K. Tomatsu N. Sato M. Roberts P. Schmidt-Erfurth U. Hitzenberger C. K., “Motion artifact and speckle noise reduction in polarization sensitive optical coherence tomography by retinal tracking,” Biomed. Opt. Express 5(1), 106–122 (2014). 10.1364/BOE.5.000106 PMC3891324 24466480

[53] Ghiglia D. C. Pritt M. D., “Two-dimensional phase unwrapping: theory, algorithms, and software,” in 1998),

[54] Desissaire S. Schwarzhans F. Salas M. Wartak A. Fischer G. Vass C. Pircher M. Hitzenberger C. K., “Analysis of longitudinal sections of retinal vessels using Doppler OCT,” Biomed. Opt. Express 11(4), 1772–1789 (2020). 10.1364/BOE.385938 32341847 PMC7173918

[55] Guizar-Sicairos M. Thurman S. T. Fienup J. R., “Efficient subpixel image registration algorithms,” Opt. Lett. 33(2), 156–158 (2008). 10.1364/OL.33.000156 18197224

[56] Matveyev A. Matveev L. Sovetsky A. Gelikonov G. Moiseev A. Zaitsev V. Y., “Vector method for strain estimation in phase-sensitive optical coherence elastography,” Laser Phys. Lett. 15(6), 065603 (2018). 10.1088/1612-202X/aab5e9

[57] Choma M. A. Ellerbee A. K. Yang C. Creazzo T. L. Izatt J. A., “Spectral-domain phase microscopy,” Opt. Lett. 30(10), 1162–1164 (2005). 10.1364/OL.30.001162 15945141

[58] Liu G. Qi W. Yu L. Chen Z., “Real-time bulk-motion-correction free Doppler variance optical coherence tomography for choroidal capillary vasculature imaging,” Opt. Express 19(4), 3657–3666 (2011). 10.1364/OE.19.003657 21369191 PMC3110778

[59] Camino A. Jia Y. Liu G. Wang J. Huang D., “Regression-based algorithm for bulk motion subtraction in optical coherence tomography angiography,” Biomed. Opt. Express 8(6), 3053–3066 (2017). 10.1364/BOE.8.003053 28663926 PMC5480449

[60] Kennedy B. F. McLaughlin R. A. Kennedy K. M. Chin L. Curatolo A. Tien A. Latham B. Saunders C. M. Sampson D. D., “Optical coherence micro-elastography: mechanical-contrast imaging of tissue microstructure,” Biomed. Opt. Express 5(7), 2113–2124 (2014). 10.1364/BOE.5.002113 25071952 PMC4102352

[61] Zhang P. Zawadzki R. J. Goswami M. Nguyen P. T. Yarov-Yarovoy V. Burns M. E. Pugh E. N., “In vivo optophysiology reveals that G-protein activation triggers osmotic swelling and increased light scattering of rod photoreceptors,” Proc. Natl. Acad. Sci. 114(14), E2937–E2946 (2017). 10.1073/pnas.1620572114 28320964 PMC5389324

[62] Azimipour M. Valente D. Vienola K. V. Werner J. S. Zawadzki R. J. Jonnal R. S., “Optoretinogram: optical measurement of human cone and rod photoreceptor responses to light,” Opt. Lett. 45(17), 4658–4661 (2020). 10.1364/OL.398868 32870829 PMC7891461

[63] Ma G. Son T. Kim T.-H. Yao X., “Functional optoretinography: concurrent OCT monitoring of intrinsic signal amplitude and phase dynamics in human photoreceptors,” Biomed. Opt. Express 12(5), 2661–2669 (2021). 10.1364/BOE.423733 34123495 PMC8176815

